# Introductory Lecture to the Course at the Medical Department of the University of Buffalo, 1900–19011Delivered at the opening exercises of the Medical Department of the University of Buffalo, September 24, 1900.

**Published:** 1900-11

**Authors:** Eugene A. Smith

**Affiliations:** Buffalo, N. Y.; Adjunct professor of clinical surgery, University of Buffalo, 1018 Main Street


BUFFALO MEDICAL JOURNAL.

VOL. XL.—LVI. NOVEMBER, 1900. No. 4

### Original Communications.

### INTRODUCTORY LECTURE TO THE COURSE AT THE MEDICAL DEPARTMENT OF THE UNIVERSITY OF BUFFALO, 1900-1901.[1]

By EUGENE A. SMITH, M. D., Buffalo, N. Y.,
Adjunct professor of clinical surgery, University of Buffalo.

THE fifty-fifth regular session of the Medical Department of the University of Buffalo begins tonight, and it has become my duty and privilege to welcome the students to the college halls, and to add such words of admonition as may tend to make them realise their responsibilities and to grasp their opportunities during the coming year. It becomes my privilege also, on this occasion, to address the public in general on matters which pertain more or less directly and intimately to the general public, as well as to the University and its teaching and student bodies.

In courtesy to the public, including the friends of the faculty and the students, some of whom are here tonight, it is my purpose to discuss first the matters which in my estimation affect the public, more especially the people of the City of Buffalo, as well as the University. While it is not my intention to minimize the importance of the Departments of Law, of Pharmacy and of Dentistry, the burden of my remarks will bear especially upon the Department of Medicine.

The people of Buffalo know the medical college as an institution which yearly brings a large number of more or less unruly students to the city and yearly adds to the present surplus of doctors. Pet cats and dogs are known to disappear during its sessions, with no relation, in the past at least, to the fear of hydrophobia, now so

1. Delivered at the opening exercises of the Medical Department of the University of Buffalo, September 24, 1900.

important a feature in the canine mortality rate. School children go around the block to avoid passing the dreadful doors of the college building, excepting high school damsels, who go a block out of their way to pass its portals when the students are not at lecture. Extremely timid people resemble the Chinese in their watchful care and anxiety for the graves of their ancestors during the college year and the continuance of dissection.

Not to elaborate this line of thought, there is to Buffalonians from another point of view a financial and material side to the presence of a large school in the city, capable, as it is, of supporting a town of fair size composed of the classes maintained by trade and subsistence. Omitting all discussion of these acknowledged financial benefits of the University, there is some controversy as to the intellectual and ethical advantages to the community resulting from the presence of the school, and it is to this point of view that I wish to give some consideration.

To me, there seems no question of its benefits in these directions. They rest upon the general proposition that a center of education is a center for culture. Theologians, physicians, jurists, statesmen, soldiers, were educated in the log cabin schools of Revolutionary times, and such schools made possible the political and social advantages we enjoy today. Ambition was enclosed by the doors of those humble schools and the scholars breathed in them the atmosphere of truth, and learned at least the one lesson of duty. Religious intolerance, superstition, injustice to the Indians and other faults may be laid at the doors of the Pilgrim forefathers, but the log school house kept the light of education burning and made possible the evolution which culminated in the Declaration of Independence.

When we compare our schools of the present with those of the past, it becomes a question whether modern advanced methods of education are doing as much for the present generation as the modern wonderful advances in methods and equipment would lead us to expect. As for the old-time medical school, maintained by the profits derived from teaching, it accomplished in the past a work of unquestioned material, intellectual and ethical benefit for the people of our country. The modern medical school in this country, where State support is not the rule, as in Europe, is in a transition stage. Some medical men, failing to perceive this, unjustly disparage their profession by proclaiming that medical teaching is stationary and pecuniary and that there are too many medical schools and too many doctors. Such expressions are at times heard from Buffalo physicians regarding

Buffalo's medical college. Some deny the steady progress of medicine or at least of medical teaching. As a matter of fact the two go hand in hand, and while all of us will admit there are too few good medical schools and too few good doctors, we can not accept the more sweeping assertions already mentioned.

It is in truth entirely correct to say that medical teaching is in process of evolution. Yearly the standard of scholarship in our medical schools is being raised. A graduate in medicine of ten years ago knows nothing of clinical pathology, more especially of hematology and bacteriology, unless he has recently taken a post-graduate course of special instruction, or has kept abreast of medical technical advances by association with the teaching body of a medical school. It argues a commercial spirit in the medical men who wish to reduce the number of medical schools and of graduates, with the intention of thereby increasing professional work for the men now in practice. If they do not advocate change from this point of view let these men bend their misdirected energies, now devoted to decrying existing schools, to the upbuilding and betterment of those schools and to raising the standard of medical education until, according to the law of the survival of the fittest, those not so fortunately situated as is Buffalo's medical college must close their doors because of lack of inducements to attract the ambitious student. Such advice, if followed, would lift the pessimistic Buffalo medical man, strongly tainted with the suspicion of commercialism, up to the plane of the medical men whose optimism leads to scientific and ethical progress in medicine. It would lift the pessimist from the trade of medicine with a purely money-earning capacity, to the profession of medicine with emulation in place of rivalry, with science placed before money. When Jenner worked out, by close observation, the value of vaccinating cowpox into the human being to immunize him against small-pox, he sacrificed the trade of medicine to the science of medicine and immortalised himself in bestowing a blessing on mankind by staying the ravages of a dreadful disease, until his time unchecked.

As regards ethics, no labored argument is needed to prove that the medical profession of a University town stands on a higher plane than that of provincial towns and cities where no such school exists. Consider the suspicion, the jealousy, the uncharitableness of the professional man toward his professional brother in country, town and city with no medical school. Professional animosities, bickerings and jealousies in Buffalo do not, and should not enter the class with them.

In our largest cities it is commercialism, not medical schools, which has led to the degeneration of medical ethics, and which has led to loud complaint and much adverse criticism. It may be admitted that where two or more rival schools struggle for existence in a territory too small for more than one, the professional tone is lower than where the demand is in just proportion to the establishment in this respect. Even here argument could be developed at length to show the ultimate evolution of ethics through a period of harsh criticism and mutual incrimination and recrimination to a time when the weary contestants, measuring themselves after the struggle, find the proper balance and relation between themselves. Given an era of such good feeling that men cease to strive to excel, that emulation should languish, and the result would be complacent mediocrity. This truth so far affects the individual that the man who has no competitors, or who far transcends all competitors, soon falls short of his best possibilities.

The relationship of the college to Buffalo must now be considered from the point of view of the faculty. Upon the faculty devolves duties and responsibilities not altogether appreciated by the public at large. Some there are who think the professorships sinecures, loaded with honors and emoluments. Few, indeed, are aware of the amount of time, the anxious thought and the physical work devoted to the shaping of the policy and the work of the college. A still smaller number know that the financial returns barely equip and run the technical and purely scientific departments of the school.

The Medical Department of the University of Buffalo had its origin in Buffalo when the city was less than a quarter of a century old. It has grown with the city for more than half a century and it has done much for the material and intellectual advancement of the city. Its present prosperity reflects a prosperous community in the surrounding country, including Western New York, Northwestern Pennsylvania, Eastern Ohio and Ontario, from which regions it draws its students. Its present aspirations, looking to the early possession of the broadest and most advanced course of the best medical schools of the country, are linked with the successful progress of the city of Buffalo toward metropolitan growth in size and wealth. Beyond any doubt the features exist which make possible the future material growth of Buffalo, and with its growth the features which go to make all the scientific and technical departments of a great university and center of learning will find a nucleus in our school. Harvard Medical School and the Boston School of Technology could not flourish simply by reason of the collegiate atmosphere of Cambridge. They had to be located in the neighboring populous city of Boston. As regards a department of art for Buffalo University a moderate degree of optimism would see a field for a strong college with Buffalo's environment and its present nucleus.

The ambitions of the faculty are deserving of earnest support from the citizens of Buffalo. Year by year the college has responded to the demands of higher medical education by improvements in its buildings and equipment and by accessions to its teaching staff. It is not of interest at this moment to repeat item by item, the gains in method and extent of teaching, as compared with a few years ago, or even with last year. This information can be found in this year's annual announcement, with which each student has made himself familiar before this. It should, however, be said that this year sees the practical outcome of the union of the medical department of Niagara University and the medical department of the University of Buffalo. This was accomplished by opening to our students the doors of the hospitals which gave fame to Niagara in years past as a college with excellent facilities for clinical teaching. Few, if any medical colleges, can command for their students the wards of so many large hospitals as does Buffalo now. With the Sisters of Charity Hospital, St. Mary's Orphan Asylum and Maternity Hospital, The Buffalo Woman's Hospital and the Providence Retreat, added to the Buffalo General Hospital, and other institutions which formerly gave the college its clinical facilities, its wealth in this respect is unexceled. In this most important feature of a medical training Buffalo overshadows most of our richest and most famous universities, owing to their location in small cities, and in contrast Buffalo's poverty in money endowment is made most glaring when compared with these same richly endowed schools. With the same endowment the University of Buffalo would occupy the position of Johns Hopkins University. It would have technical and scientific schools which for many years at least would by virtue of environment overshadow the departments of arts, of theology and of law.

Possessing unexceled, if not unrivaled opportunities for scientific research and instruction in her wealth of clinical material, the University of Buffalo has also in her teaching body men imbued with the scientific spirit. The home of the medical college was very humble in its infancy, yet James P. White, Julius F. Miner, Frank H. Hamilton, Austin Flint, John C. Dalton, Edward M. Moore and Thomas F.

Rochester were giants in the history of medicine in the past, who dignified the frame work of wood and stone, called the old Buffalo Medical College, with the fame of their deeds and names. Buffalo between 1846 and 1870 is of interest to the antiquarian in old prints and photographs, but it is of interest to the present generation much more by reason of the deeds of such men as were just named, and in 1950, beyond a doubt, Buffalo of 1900 will be remembered by the deeds and names of men now in the harness in the heat and the toil of the various walks of life. In the literary and scientific fields, men whom we now daily watch, praise, admire, imitate, criticise, blame, misunderstand, misjudge, and even condemn or despise, will stand in proper perspective before the coming generation, some of them giants in intellect as were the men who founded our college in the decade 1845 to 1855.

Without thought of servility or flattery, we may claim for the university scientific men imbued with the spirit and possessing the ability so to discharge their duties as teachers of students and as leaders of medical thought, that the future will more fittingly assign them their just reward of fame and position than we of the present can do, influenced as we are by the personal considerations, which in the frailty of human nature warp our judgments by limiting our view of the characters and acts of our contemporaries.

The University of Buffalo belongs to Buffalo. It alone lifts Buffalo into the atmosphere of literary and scientific activity, to which commercial and material prosperity have given the city a right to aspire. It has a field and a mission. Its faculty has the welfare of the school at heart, but endowment is necessary to enable the departments to unfold as they could and should.

When we read of the sums given to the cause of education we find how Buffalo suffers in comparison. About $30,000,000 were given last year, mostly to the large colleges already rich. Of this sum more than half was given by donors who are still living to observe with mortal eyes the good their gifts are doing. This should point a moral to some philanthropic citizen of Buffalo, or resident of the surrounding country. He can raise a monument to his memory and live to be a spectator of its benefits by generously endowing those departments of the college, that may render incalculable service to mankind in the unknown fields of science. Where such endowment can be obtained only as a bequest, its usefulness is seriously impaired and may be indefinitely delayed. The legal profession is prone to develop some complication in a will, which, medically speaking, makes the prognosis as regards benefit to the college exceedingly dubious. An irritated professor whose college was thus kept starving in sight of the promised land of plenty was moved to write the following song:

"God bless you, Gentlemen ! Learn to give
Money to colleges while you live.
Don't be silly and think you'll try
To bother the colleges when you die,
With codicil this, and codicil that,
That Knowledge may starve while Law grows fat ;
For there never was pitcher that wouldn't spill,
And there's always a flaw in a donkey's will."

It may be possible the printer erred in saying "a donkey's will," and that the professor wrote it "donor," but the verse shows it donkey.

In the past few weeks a matter of endowment has been developing of extreme interest to all who have faith and pride in our school. The sum of $25,000 was given by one generous donor, whose name is still withheld, and others adding to it, a sum of nearly $40,000 is now available, under college management, for building and equipping an institute which is to be primarily designed to prosecute the research work in cancer now being conducted. The scope of the work, however, is so elaborate that room will be found for research in all of the different branches of medical science. The plans provide for rooms devoted to research in bacteriology, pathology, physiological chemistry, microscopy, microphotography and radiography. The equipment in these various branches of science will be complete and the opportunity for original work in the great field of experimental research will be inferior to no laboratory. What new discoveries, rivaling those of Pasteur in scientific interest, and what new practical benefits may thereby be gained for the world, scientific laboratories are best fitted to reveal. Let us hope for Buffalo that some of the honors and laurels may be won by our workers in these fields.

It is hoped that the building can be completed and put into use by next Spring. It will be near the college in the immediate vicinity of the General Hospital, and thus advantageously placed for scientific work, will mark the first great stride toward placing the University of Buffalo on an equal footing with the best institutions of learning in New York, Philadelphia and Baltimore.

This endowment is of interest from another point of view. New York State's government and philanthropy are here joined in furthering scientific work. The dread of cancer and of its tendency to a fatal termination has led the legislature of New York to appropriate money generously to investigate the disease with a view to ultimate curative discoveries. Buffalo Medical College has given a home to the state laboratory. In Europe such enterprises, as well as the universities, are entirely supported by the state. In this country, as institutions of learning have outgrown the stock company period of development and have become more worthy of existence, philanthropic gifts and bequests have in the main been the source of revenue and support. With state aid and philanthropy now united in furthering one branch of our medical college, how ungracious in the future will be any carping criticism of the worthy aims and ambitions of the University or its faculty on the part of any Buffalonian! If all rally to the support of our school, working unitedly with singleness of purpose, it will soon obtain the highest confidence of the educational world, and will attract men who will add to the city's fame. Richer schools in the past have taken from us good men in literary and scientific lines, attracting them to broader fields than ours. Let the near future show us the way to bring them back and others with them, if we can not here develop the talent we no doubt have among us!

To the medical profession in Buffalo and the vicinity, let me recommend a discussion of any differences we may have on medical and educational matters among ourselves, thus promoting our united action and professional dignity, and disarming the criticism and distrust on the part of the laity which a free public discussion of such differences must induce.

When I now turn to address the students, I take pleasure in extending to all the classes a cordial welcome on behalf of the faculty. Taking it for granted that you all have entered the college with the ambition to make the study and practice of medicine in the broad sense of medical science your life-work, I congratulate you. Next to the service of the church you have chosen, in my estimation, the noblest profession. The duties, possibilities and rewards of the profession of medicine it is more fitting to discuss in a valedictory address at commencement, rather than now at the opening exercises of the college year. Nor do I wish to recapitulate the advantages in the features of the course, as laid down in the curriculum which Buffalo Medical College has to offer, to make the year 1900-1901 a notably auspicious year. Words of admonition will not, I trust, be taken to indicate that I consider students lacking in system in their work, diligence in its pursuit or steadfastness to carry it to completion. Rather let me speak as one who was a matriculant in medicine some few years ago. The young men and women who sit before me, about to resume their work, or who appear here to begin their career in medicine, have but a faint idea of the increase in knowledge, and the increase in ease of acquisition of that knowledge with instruments of precision, as compared with the course in medicine which their teachers were given.

Medicine as a science is still progressing, and you are also students in its expanding field. Fully to grasp the meaning of facts now known to us all and to deduce therefrom truths still to be discovered, would mean fame and fortune almost at once upon graduation. Begin and continue diligent, persevering and systematic study. As a freshman do not fritter away time in the thought that with four years of time, work can be begun in earnest at some later date. As a sophomore, build upon your foundation of elementary work the beams of the superstructure of practical medicine. As a junior complete this framework. As a senior, with commencement less than a year ahead, opening to your view the prospect of personal responsibility in the competition of life, seek to embellish the framework of practical medicine with its crowning decoration, a judgment founded upon cultivated and trained powers of observation. Seek to rise above medicine as a "science of guesswork" as some one has reproachfully called it. Say but little of exploratory operations to make diagnoses. Disregard advice to give medicines on the ground that if they do no good they can do no harm. In the laboratory, study methods and reasons rather than merely to copy routine experiments. At the clinic observe closely a demonstration. See, feel and hear what the instructor points out, and reason on his reasoning. It is not the ability to say I have seen a case of hydrophobia, but the ability to diagnosticate a case of hydrophobia from one of hysteria, which makes the medical man. Upon examining a patient, how often is disease found in various parts of the human mechanism! To select and treat the organ most essential to life, and then to treat in their order such organs as need treatment to restore health marks the ideal practitioner.

Classify and systematise your facts, avoiding collecting them hap-hazard, thus developing your judgment. This advice is exemplified in the story of the pilot on one of our great rivers, who was asked by a young man how long he had been a pilot. He answered: "twenty five years." Then," said the young man, "I suppose you know every rock and every sand-bar in this river." "Oh, no! I don't," said the old pilot, "but I know where the deep water is."

The pilot's wisdom corresponds to the experience of the old family physician. The methods and explanation of the young doctor are more admired, but the old man's advice is more trusted. When we say a young man has an old head, we mean he has early developed the judgment that men ordinarily take years of experience to acquire.

In conclusion, I could wish for some small inspiration of the gift of prophecy to address public, faculty and students on so auspicious an occasion as the opening of the college year 1900-1901, closing as it does the nineteenth century and beginning the twentieth. The fortune of the University of Buffalo has kept pace in the past with the remarkable growth of Buffalo. We all look confidently for continued prosperity for Buffalo, and in the coming year, especially with the Pan-American Exposition, now unfolding so promisingly, the University should develop in a degree commensurate with the development of Buffalo. Let the medical profession educate the public to believe in the college and we can certainly hope, if we can not actually prophecy, that the needs of the University of Buffalo will soon be met by an adequate endowment, enabling it to continue in the future its career of usefulness to man and of honor to the city—the center for Buffalo of intellectual and ethical culture.

1018 Main Street.

